# Clinical Characteristics of Pediatric Esophagitis in Southern Iran; A Single-Center Experience

**Published:** 2013-06

**Authors:** Mozhgan Zahmatkeshan, Khadijesadat Najib, Bita Geramizadeh, Ebrahim Fallahzadeh, Mahmood Haghighat, Mohammad Hadi Imanieh

**Affiliations:** 1 Department of Pediatric Gastroenterology, Nemazee Hospital, Shiraz University of Medical Sciences, Shiraz, Iran;; 2 Department of Pediatric, Nemazee Hospital, Shiraz University of Medical Sciences, Shiraz, Iran;; 3 Department of Pathology, Shiraz Transplant Research Center, Nemazee Hospital, Shiraz University of Medical Sciences, Shiraz, Iran;; 4 Department of Internal Medicine, Nemazee Hospital, Shiraz University of Medical Sciences, Shiraz, Iran

**Keywords:** Esophagitis, Pediatrics, Reflux esophagitis, Eosinophilic esophagitis, Iran

## Abstract

**Background: **We sought to determine the clinical characteristics of pediatric esophagitis in southern Iran.

**Methods: **This cross-sectional study was conducted over a 4-year period, from 2005 to 2009, in Nemazee Hospital, a tertiary healthcare center in Shiraz, southern Iran. We consecutively included all pediatric patients (<18 years) who underwent endoscopy in our center and had pathology-confirmed diagnosis of esophagitis. Data regarding the patients’ demographic characteristics, comorbidities, and clinical findings were recorded using a questionnaire. All the patients underwent upper gastrointestinal endoscopy and biopsy of the esophagus, and the findings were recorded in the questionnaire.

**Results: **We studied 125 children, comprising 61 (48.8%) girls and 64 (51.2%) boys at a mean age of 6.6±5.5 years. Repeated vomiting was the prominent symptom in our series, with it being reported by 75 (60%) patients, followed by fever in 35 (28%). Erythema (33.6%), esophageal ulcer (11.2%), and whitish patch (8.0%) were the most common endoscopic findings, while reflux esophagitis (32.8%), chronic (6.4%) and acute esophagitis (5.6%), and candida esophagitis (5.6%) were the most common histological diagnoses. Only one (0.8%) patient was diagnosed as having eosinophilic esophagitis, aspergillosis, and graft-versus-host disease.

**Conclusion: **Reflux was the most common cause of esophagitis in the pediatric population of southern Iran. Contrary to previous reports, the prevalence of eosinophilic esophagitis was far less than that estimated, while the prevalence of opportunistic infections was higher secondary to post-liver transplantation immunosuppression.

## Introduction

Esophagitis is the inflammation of the squamous esophageal epithelium and may occur in early life, even in infancy, when it may be difficult to be differentiated from infantile colic.^1^ Gastroesophageal reflux disease (GERD) is the most common etiology of esophagitis in the pediatric age group. Other important, albeit less common, types of esophagitis in children are eosinophilic esophagitis, allergic esophagitis, infectious esophagitis in immunocompromised patients, radiation esophagitis, and erosive esophagitis from ingestion of corrosive agents such as certain medications or cleaning products.^2^

Children with esophagitis usually have various non-specific signs and symptoms. The clinical presentation depends on the etiology and the patient’s age. Those infants and children with GERD-induced esophagitis may have a history of repeated vomiting, irritability, and respiratory problems along with progressive symptoms such as difficulty in swallowing and inconsolable crying. The cases of corrosive esophagitis may be associated with life-threatening signs such as bleeding, upper airway obstruction, and hemodynamic compromise.^3^ Whatever the underlying cause, if left untreated, all types of esophagitis may be complicated by strictures. Generally in children, the clinical diagnosis of esophagitis tends to become very complicated by many differential diagnoses, from self-limited gastrointestinal disorders to emergency cases. A few paraclinical tests are helpful for the diagnosis of esophagitis, notably in children, and the definite diagnosis relies on esophagogastroduodenoscopy and histological findings.^4^

Although symptomatic treatment can be considered for all children with esophagitis, specific treatment plans depend on the underlying etiology. Because of the differences in socio-demographic characteristics such as child care, environmental exposures, and hygienic status, the etiology of esophagitis may vary in different populations. Furthermore, those with immunodeficiency or those receiving radiation therapies are at high risk of esophagitis. The present study, one of the scarce reports from developing countries, aimed to determine the clinical characteristics of pediatric esophagitis in southern Iran.

## Patients and Methods

This cross-sectional study was conducted over a 4-year period (from 2005 to 2009) in Nemazee Hospital, a tertiary healthcare center affiliated to Shiraz University of Medical Sciences. (Nemazee Hospital is a referral center for five southern Iranian provinces.) We consecutively included all pediatric patients (<18 years) who underwent endoscopy in our center and had pathology-confirmed diagnosis of esophagitis. Patients with suspicious or equivocal diagnoses as well as those with incomplete reports and/or demographic features were excluded from the study. 

The study protocol was approved by the Institutional Review Board and Ethics Committee of Shiraz University of Medical Sciences. In keeping with all medical procedures for the pediatric age group, verbal and written informed consents were obtained from the patients and their parents or legal guardians, respectively.

All the patients were initially visited by a pediatrician. Data regarding the patients’ demographic characteristics, comorbidities, and clinical findings were recorded using a questionnaire. All the patients underwent upper gastrointestinal (GI) endoscopy with a small, flexible Olympus (Melville, NY) GIF-N30 endoscope by four pediatric gastroenterologists in the Pediatric Gastroenterology Department of our center, and the findings were recorded in the questionnaire. 

The patients received Midazolam (1mg/kg) intravenously several minutes before endoscopy as a sedative agent. Several mucosal biopsies were taken under direct visualization of the mucosa and the vasculature. Phosphate-buffered formalin was used for fixing the specimens and biopsies. Paraffin-embedded specimens were sectioned in 5-6 µm slices and were further stained with hematoxylin and eosin according to the standard laboratory methods. All the slides were reviewed by a pathologist, and the results were recorded in the questionnaire. The criteria for reflux esophagitis were comprised of basal zone hyperplasia, elongated stromal papillae, and vascular ingrowths.

The drugs consumed by the patients were further categorized as those being unrelated to esophagitis, those being responsible for inducing esophagitis, and those being effective in the treatment of esophagitis. NSAIDs, Prednisolone, Mycophenolate Mofetil (CellCept), Cyclosporine, Acyclovir, Metronidazole, Meropenem, Cyclophosphamide, Methotrexate, Warfarin, Ciprofloxacin, Erythromycin, Co-trimoxazole, Fluconazole, Mesalamine, and Tacrolimus were considered to be responsible for inducing esophagitis, whereas Omeprazole, Pantoprazole, Metoclopramide, Ondansetron, Ranitidine, Cimetidine, aluminium-magnesium, and Motilium were considered as effective agents in the treatment of esophagitis.

The data were prospectively entered into a computer database, and were further analyzed by SPSS software, version 14.0 (SPSS Inc., Chicago, Illinois, USA). The data are reported as mean±SD or proportions.

## Results

We studied 125 children with pathology-confirmed esophagitis. The study population consisted of 61 (48.8%) girls and 64 (51.2%) boys at a mean age of 6.6±5.5 years. The demographic and clinical characteristics of the patients are summarized in table 1. 

**Table 1:** Demographic and clinical characteristics of 125 pediatric patients with esophagitis

**Variable **	**Value **
Age (years)	6.6±5.5
Sex	
Girls (%)	61 (48.8%)
Boys (%)	64 (51.2%)
Nutrition	
>2 years of age (%)	75 (60%)
Breastfed with eliminating dairy (%)	16 (12.7%)
Breastfed (%)	14 (11.2%)
Milk-based formulas (%)	13 (10.4%)
Soy-based formulas (%)	7 (5.6%)
Clinical signs and symptoms	
Repeated vomiting (%)	75 (60%)
Fever (%)	35 (28%)
Failure to thrive (%)	24 (19.2%)
Dysphasia (%)	22 (17.6%)
Bloody vomiting (%)	18 (14.4%)
Agitation (%)	9 (7.2%)
Drug history	
Agents responsible for esophagitis (%)	107 (85.6%)
Agents unrelated to esophagitis (%)	18 (14.4%)
Comorbidities	
Liver transplantation (%)	48 (44.9%)
Thrombocytopenia (%)	29 (23.4%)
Prolonged prothrombin time (%)	16 (16%)
Malignancy (%)	14 (11.2%)
Prolonged partial thromboplastin time (%)	11 (8.8%)
Autoimmune or immunodeficiency (%)	10 (8%)

Repeated vomiting was the prominent symptom in our series, which was it being reported by 75 (60%) patients, followed by fever in 35 (28%) and failure to thrive in 24 (19.2%). Most of the patients (60%) were more than 2 years of age and, thus, consumed a large variety of food. However, 16 (12.7%) patients were breastfed with a dairy elimination diet, while 14 (11.2%) were breastfed. The drugs being consumed by 107 (85.6%) patients were found to be responsible for inducing esophagitis, whereas in the others (14.4%), the drugs were unrelated to the disease. Liver transplantation (44.9%) and thrombocytopenia (23.4%) were the most common related comorbidities. 

The endoscopic and pathological findings are depicted in table 2. Most of the patients (38.4%) had normal endoscopic findings. Erythema (33.6%), esophageal ulcer (11.2%), and whitish patch (8.0%) were the most common endoscopic findings of the patients. Histological examination revealed non-specific findings in most of the patients (33.6%). Nevertheless, reflux esophagitis (32.8%), followed by chronic (6.4%) and acute esophagitis (5.6%) and candida esophagitis (5.6%), was the most common histological diagnoses. Only one (0.8%) patient was diagnosed as having eosinophilic esophagitis, aspergillosis, and graft versus host disease. All the patients received appropriate treatment; and except for 14 (11.2%) patients, the rest were followed up for evaluation until the end of the study period. Almost all the patients (73.6%) were asymptomatic in a 6-month period after diagnosis, while some (6.4%) had chronic disease without improvement. 

**Table 2:** Endoscopic and pathology findings of 125 pediatric patients with esophagitis

**Variable **	**Value **
Endoscopy findings	
Normal (%)	48 (38.4%)
Erythema (%)	42 (33.6%)
Ulcer (%)	14 (11.2%)
Whitish patch (%)	10 (8.0%)
Structural abnormalities (%)	5 (4.0%)
Erosion	4 (3.2%)
Nodularity (%)	2 (1.6%)
Pathology findings	
Non-specific (%)	42 (33.6%)
Reflux esophagitis (%)	41 (32.8%)
Chronic esophagitis (%)	8 (6.4%)
Acute esophagitis (%)	7 (5.6%)
Candida esophagitis (%)	7 (5.6%)
Herpetic esophagitis (%)	4 (3.2%)
Esophageal stenosis (%)	3 (2.4%)
Lymphoid nodular hyperplasia (%)	2 (1.6%)
CMV esophagitis (%)	2 (1.6%)
Infective esophagitis (%)	1 (0.8%)
Aspergillosis (%)	1 (0.8%)
Hyperplastic squamous mucosa (%)	1 (0.8%)
Eosinophilic esophagitis (%)	1 (0.8%)
Lymphoma (%)	1 (0.8%)
GVHD (%)	1 (0.8%)
Congestive esophagitis (%)	1 (0.8%)
Leiomyoma esophagitis (%)	1 (0.8%)
Chemotherapy-induced esophagitis (%)	1 (0.8%)

CMV: cytomegalovirus; GVHD: graft-versus-host disease

## Discussion

The prevalence of esophagitis in the pediatric population has increased in the recent decade, mostly because of the increase in the incidence of GERD in children.^5^ However, this escalating trend might be, at least in part, in consequence of a rise in the diagnosis of this disorder. Although several studies have investigated the pattern of pediatric esophagitis,^2^^-^^5^ data regarding this issue in our region are scarce. We found that most of our pediatric patients with esophagitis were more than 2 years of age. Repeated vomiting was the prominent symptom in our series, and liver transplantation was the most common related comorbidity. The most common endoscopic and histological findings were erythema and reflux esophagitis, respectively. 

The prevalence of pediatric esophagitis is largely unknown. In this regards, Gilger and colleagues,^7^ found the prevalence of erosive esophagitis to be 12.4% in a population of 888 pediatric patients referring to Texas Pediatric Medical Center. The mean age of the patients in that study was 12.7±4.9 years, which is extremely higher than that in our study. Similarly, the prevalence of esophagitis in children suffering from upper digestive complaints was reported by Rafeey and Ghatami,^6^ to be 82.9% in a sample of Iranian population. The authors also reported that the most common age group for pediatric esophagitis was 8-12 years, which is very different from that in our series. 

Gill and colleagues,^8^ conducted a cross-sectional study, including 1424 diagnostic upper endoscopies, between 1995 and 2004, and reported a prevalence rate of 0.73/10,000 for eosinophilic esophagitis in children. The authors found a higher prevalence in older age groups compared with ours. The possible explanation for this age discrepancy can be the fact that the most common etiologies of esophagitis in our series were liver transplantation and postoperative immunosuppression. 

In our study, the most frequent symptoms of esophagitis were vomiting and fever. Our findings are consistent with those of another study, in which vomiting was the most frequent symptom followed by abdominal pain and cough.^9^ Another report also showed that abdominal pain and vomiting were the presenting symptoms of esophagitis in Iranian children.^6^

Symptoms of GERD are reported in 2-7% of children. The clinical feature can be limited to symptoms such as heartburn and regurgitation, or can be complicated with erosive esophagitis, esophageal strictures, or Barrett esophagus.^7^ Symptoms of eosinophilic esophagitis mimic GERD. This type of esophagitis is an allergic inflammatory reaction. To differentiate between GERD and esophagitis, histological confirmation is necessary.^10^ A new definition proposes that eosinophilic esophagitis is a chronic, immune/antigen-mediated disease, which is diagnosed by both clinical and pathological features.^11^ Almost all the previous reports show that reflux esophagitis is the most common type in pediatric patients ranging from 10.3%,^2^ to 56.8%.^7^ This is consistent with our findings, according to which reflux was responsible for 32.8% of cases. Be that as it may, we presume that the prevalence of reflux esophagitis is higher because our study included only those pediatric patients who were resistant to medical treatment or had acute presentations such as upper GI bleeding, while many patients with reflux esophagitis are treated medically in an outpatient setting without undergoing endoscopy. 

In children, eosinophilic esophagitis is mostly a food-hypersensitivity disorder. Treatment with the standard food elimination diet, i.e. diet excluding cow’s milk protein, soy, wheat, egg, peanut, and seafood, is usually successful.^12^^-^^14^ Many food proteins can act as antigens in humans. Cow’s milk proteins are most frequently considered as a cause of food intolerance during infancy. It can be associated with GERD and esophagitis.^15^ The prevalence of eosinophilic esophagitis has been reported to range from 0.73/10,000,^8^ to 52/100,000,^16^ and the trend has been described to be increasing.^17^ Nonetheless, we had only one (0.8%) patient with eosinophilic esophagitis, which is lower than that in the previous reports. 

Most of our patients, who were resistant to medical therapy, had received different forms of formula or dairy eliminated milk based on allergic or eosinophilic esophagitis diagnosis, while only 2 (1.6%) patients had lymphonodular hyperplasia and one (0.8%) eosinophilic esophagitis. Further studies are needed to investigate the prevalence of milk allergy in the Iranian population. 

A high proportion of our patients suffered from opportunistic infections, including candida, aspergillosis, cytomegalovirus, and herpes. This is consistent with the most common comorbidity in our study, which was liver transplantation. Our center is the only center for liver transplantation in Iran and, thus, postoperative immunosuppression is the most common risk factor for esophagitis in the pediatric population. The high prevalence of opportunistic infections can be attributed to the whole country because all the patients are referred to our center from all over Iran.

## Conclusion

Reflux was the most common finding in our pediatric population with esophagitis in southern Iran. Contrary to the previous reports, the prevalence of eosinophilic esophagitis was far less than that estimated, while the prevalence of opportunistic infections was higher secondary to post-liver transplantation immunosuppression. Further studies are required to investigate the prevalence of allergic esophagitis in southern Iran. 

## References

[1] Pruvost I, Aubry E, Martinot A (2011). [Diagnosis of acute abdominal pain in infants]. Rev Prat.

[2] Dahms BB (2004). Reflux esophagitis: sequelae and differential diagnosis in infants and children including eosinophilic esophagitis. Pediatr Dev Pathol.

[3] Hassall E (2008). Step-up and step-down approaches to treatment of gastroesophageal reflux disease in children. Curr Gastroenterol Rep.

[4] Putnam PE, Rothenberg ME (2009). Eosinophilic esophagitis: concepts, controversies, and evidence. Curr Gastroenterol Rep.

[5] Thomas A (1997). Paediatric Gastrointestinal Disease. Pathology, Diagnosis, Management. Arch Dis Child.

[6] Rafeey M, Ghatami G (2004). P1025 Incidence and Epidemiology of Esophagitis in Children Admitted to Endoscopy Unit in Medical Center of Children in Tehran. Journal of Pediatric Gastroenterology and Nutrition. Journal of Pediatric Gastroenterology & Nutrition.

[7] Gilger MA, El-Serag HB, Gold BD, Dietrich CL, Tsou V, McDuffie A (2008). Prevalence of endoscopic findings of erosive esophagitis in children: a population-based study. J Pediatr Gastroenterol Nutr.

[8] Gill R, Durst P, Rewalt M, Elitsur Y (2007). Eosinophilic esophagitis disease in children from West Virginia: a review of the last decade (1995-2004). Am J Gastroenterol.

[9] Gupta SK, Hassall E, Chiu YL, Amer F, Heyman MB (2006). Presenting symptoms of nonerosive and erosive esophagitis in pediatric patients. Dig Dis Sci.

[10] Hopp RJ (2012). Eosinophilic Esophagitis in Pediatrics: The Worst of all Possible Allergy Worlds. J Allergy (Cairo).

[11] Liacouras CA, Furuta GT, Hirano I, Atkins D, Attwood SE, Bonis PA (2011). Eosinophilic esophagitis: updated consensus recommendations for children and adults. J Allergy Clin Immunol.

[12] Kagalwalla AF, Sentongo TA, Ritz S, Hess T, Nelson SP, Emerick KM (2006). Effect of six-food elimination diet on clinical and histologic outcomes in eosinophilic esophagitis. Clin Gastroenterol Hepatol.

[13] Berni Canani R, Di Costanzo M, Troncone R (2011). The optimal diagnostic workup for children with suspected food allergy. Nutrition.

[14] Kagalwalla AF, Shah A, Li BU, Sentongo TA, Ritz S, Manuel-Rubio M (2011). Identification of specific foods responsible for inflammation in children with eosinophilic esophagitis successfully treated with empiric elimination diet. J Pediatr Gastroenterol Nutr.

[15] Nielsen RG, Bindslev-Jensen C, Kruse-Andersen S, Husby S (2004). Severe gastroesophageal reflux disease and cow milk hypersensitivity in infants and children: disease association and evaluation of a new challenge procedure. J Pediatr Gastroenterol Nutr.

[16] Spergel JM, Book WM, Mays E, Song L, Shah SS, Talley NJ (2011). Variation in prevalence, diagnostic criteria, and initial management options for eosinophilic gastrointestinal diseases in the United States. J Pediatr Gastroenterol Nutr.

[17] Gupte AR, Draganov PV (2009). Eosinophilic esophagitis. World J Gastroenterol.

